# Aortic elasticity changes in thalassemia due to heart and liver iron deposition

**Published:** 2023

**Authors:** Noor Mohammad Noori, Alireza Teimouri

**Affiliations:** 1 Children and Adolescents Health research center, Research Institute of cellular and Molecular Science in Infectious Diseases , Zahedan University of Medical Sciences, Zahedan, Iran; 2 Children and Adolescent Health Research Center, Resistant Tuberculosis Institute, School of Medicine, Zahedan University of Medical Sciences, Zahedan, Iran

**Keywords:** Aortic elasticity, Iron overload, Heart, Liver, Thalassemia

## Abstract

**Background:**

Iron overload is connected with an expanded prevalence of thalassemia due to heart impairment. This considers pointing to survey changes in thalassemia's aortic elasticity due to iron deposition in the heart and liver of children.

**Methods:**

This case-control study was performed on 80 healthy and 160 thalassemia patients. The subjects gathered from educational pediatric hospital of Ali Asghar in Zahedan, Iran, from 2019 to 2021. Echocardiography parameters were measured. Ferritin, lipids profile, cardiac and liver MRI T2 * measured in patients only. Aortic elasticity parameters were aortic strain, aortic stiffness β index, aortic distensibility and pressure strain elastic modulus. Data analyzed by SPSS,p< 0.05 was considered as significant.

**Results:**

Diastolic blood pressure (p<0.001), aortic diameter in diastole (p<0.001), aortic diameter in systole (p<0.001), ferritin (p<0.001), aortic strain (p<0.001), aortic distensibility(p<0.001), pressure strain elastic modulus (p<0.001) and aortic stiffness β index (p<0.001) were changed significantly in thalassemia patients compared to controls. From these variables, AoD, AoS, ferritin, AS and AD increased in thalassemia. Ferritin was higher in thalassemia patients with abnormal heart iron deposition (2131.89±1992.74 v.s 4887.66±3122.59 ng/ml). Considering the level of liver iron deposition, ferritin did not change in patients. Our highlighted variables did not change in patients based on the groups of ferritin.

**Conclusion:**

Concluded that AoD, AoS, ferritin, AS and AD increased in thalassemia patients. Ferritin increased in thalassemia with abnormal iron overload in the heart but did not change in the liver. Recommended MRI T2* to evaluate dynamic functions of liver and heart in thalassemia patients.

Thalassemia is a blood disorder that was inherited from parents and characterized by abnormal hemoglobin (1, 2), more frequented in Mediterranean Sea area (3,4). Iran has approximately 25,000 thalassemia patients with three million carriers (1). Sistan and Baluchestan province in Iran has thalassemia gene frequency in high numbers of patients (5).

 Iron overload is connected with expanded occurrence of transfusion-dependent (TDT) and non-transfusion-dependent thalassemia (NTDT) (5,6). The main mechanisms causing the iron loading process is due to transfusion at TDT and increased intestinal absorption due to ineffective erythropoiesis and hepcidin suppression in NTDT (7). In thalassemia, iron overload that measured by ferritin, causes systolic and diastolic dysfunction of the left ventricle in the myocardium as a main cause of mortality and it does not occur until the other organs are saturated (8-10). The most important organs affected by iron overload are heart and liver (9).

Between the liver and heart, ferritin level is poorly correlated with heart iron overload compared to liver that this overload can be measured by MRI. Iron overload is caused by increased iron deposition in the human body and emerges as an important cause of congestive heart failure more common in thalassemia (11), and among the methods to assess iron deposition in body organs, the T2 * method is more sensitive to quantification and longitudinal tracking (12).

Cardiac MRI T2 * has been proven to be quick and simple and robust with high positive value ​​to predict cardiac dysfunction in asymptomatic thalassemia (13) such as changes in arterial stiffness (9) that reported and evaluated by several documents (8,9,14). According to the mechanisms mentioned above and because the iron overload has negative impact on aortic elastic function, the present study aimed to assess the variation of stiffing in thalassemia due to iron overload in heart and liver.

## Methods

Study Design: This case-control study was performed on 240 participants aged 5 to 39 years consisted of 80 healthy and 160 thalassemia as case group in Ali Asghar Hospitals of Zahedan in Sistan and Baluchistan provinces, Iran, from 2019 September to 2021 August. This study enrolled thalassemia patients who were asymptomatic 48-72 hours after packed red blood cell transfusions and received regular hemoglobin transfusions above 10 g / dl. Of the patients, only 80 people had heart and liver MRI T2 * due to iron deposits. Sampling of the controls was hospital base from those referred for the routine examinations.

Criteria: Participants invalvular heart disease, rhythms, structural abnormalities, active infections, systemic inflammatory diseases, and renal failure were excluded from the study.

**Measurements**

**Blood Pressure: **Blood pressure (BP) levels were measured from the brachial artery at the level of the heart with a sphygmomanometer after resting for at least 5 minutes in the supine position. Three measurements, at least 2-minute apart, were performed, and the average of the closest two readings was recorded. A pressure drop rate of approximately 2 mm Hg/s was applied, and Korotkoff's phases I and V were used for systolic and diastolic BP levels. All BP measurements were made by a cardiologist. 

**Echocardiography: **The patient's important procedures were medical history, physical examination, chest x-ray, and echocardiography by a designated cardiologist. Echocardiography was performed on participants by the cardiologist using My Laboratory 60 with Transducer 3, 8 (made in Italy). To reach high accuracy of echocardiographic findings, the measurements were repeated 3 cycles and the average was taken into account. Participants underwent echocardiography without holding their breath. 

Echocardiographic results from M-mode (a diagnostic ultrasound representation of changes in echo over time indicating the depth of the echogenic interface with two-time axes and motion axes) are the diastolic diameter (AOD) of the aorta, the aortic. It was systolic diameter (AOS). The diameter of the aorta was measured as the distance between the inner edges of the anterior and posterior walls of the systolic and diastolic aorta. AOS was recorded when the aortic wall was fully opened. AOD was recorded at the same time that the QRS complex was seen on the electrocardiogram (ECG) recording. The measurements were made between three consecutive pulses and the average was calculated.

**Cardiac and liver MRI: **To measure myocardial T2 *, standard ECG gating was used to synchronize scans with the cardiac cycle. Next, we took one short axis with a thickness of 10 mm, which was placed between the base of the left ventricle and the apex of the heart. Liver MRIT2 * was measured by imaging a single longitudinal slice (10 mm) through the center of the liver. Cardiac MRI T2 * results were categorized into severe (T2 * <10ms), moderate (10 <T2 * < 14 ms), and mild (14 < T2 * < 20> 20 ms) myocardial lesions. The results of liver T2* where acceptable liver iron was defined as LIC <3.5 mg/g, while mild, moderate, and severe were 3.5–7.0, 7.0–12.0, and >12.0, respectively (15).

**Serum ferritin level: **From participants, 3 ml of blood was taken by a nurse at 8:00 am. Samples were centrifuged at 3000 g for 10 minutes at 5 ° C. Sep -70 fridges until measurement time of ferritin and leptin. Finally, under the cold chain, the samples were transferred to the Biochemistry Lab of Zahedan University of Medical Sciences (ZaUMS). Then, 250 microns was isolated from serum samples to analyze ferritin by ELISA method/kit (USA).

**Figure 1:**
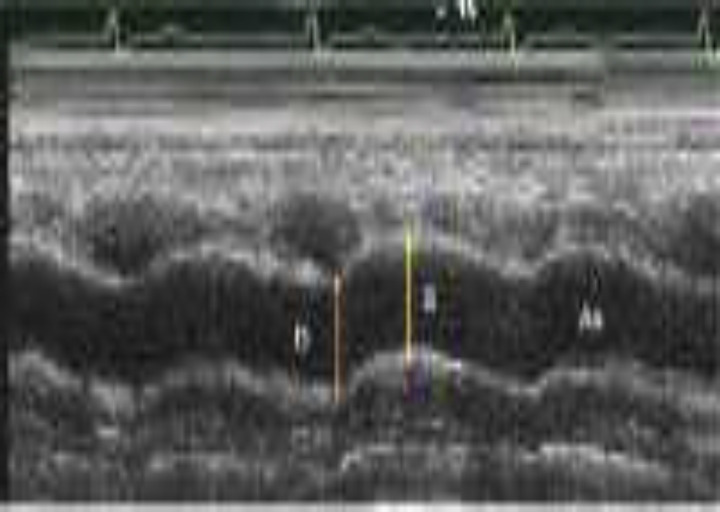
Measurements of aortic diameter in systole (S) and diastolic (D) of the ascending aorta in M-mode tracing obtained at a level 3 cm above the aortic valve (9)

**Aortic Elasticity Parameters: **The systolic and diastolic diameters of the ascending aorta were recorded in M ​​mode under the guidance of echocardiography and electrocardiography, approximately 3 cm above the aortic valve from the parasternal long axis. The systolic diameter of the aorta was measured at the time of maximal aortic advance, and the diastolic diameter was measured at the start of the QRS complex by electrocardiography (figure 1). The aortic elasticity of the aorta was evaluated using the following equation (16). Aortic strain (%) = (aortic diameter in   systole − aortic diameter in diastole) × 100/aortic diameter in diastole. Aortic stiffness β index = Ln (systolic blood pressure / diastolic blood pressure)/ ([aortic diameter in   systole − aortic diameter in diastole]/ aortic diameter in diastole). Aortic distensibility (cm2 × dyne-1.10–6) = 2 × ([aortic diameter in   systole − aortic diameter in diastole]/ aortic diameter in diastole)/ (systolic blood pressure -diastolic blood pressure). Pressure strain elastic modulus = (systolic blood pressure -diastolic blood pressure)/([aortic diameter in systole – aortic diameter in diastole]/ aortic diameter in diastole).

**Anthropomorphic Measurements: **Participant’s height and weight were measured by experienced expert using standard equipment. Next, BMI was calculated as weight (kg) / height (m2).

**Ethical Approval: **After approval, an informed consent was obtained from the participants or their legal guardians. This study was approved as a project (approved by the Research Commission and numbered 9397) and approved by the Ethics Commission of Zahedan Medical University in Zahedan, Iran (Ethics Number: IR.ZAUMS. REC.1398.410).

**Statistical Analysis: **To analyze data, SPSS 20.0 (SPSS Inc.; Chicago, Illinois, United States) applied. To assess distribution of continuous variables Kolmogorov–Smirnov test was used. Student’s t-test and Mann–Whitney U-test were applied to compare two mean values of quantitative variables with normal and non-normal variables. In comparing the variables in three or more groups, one-way analysis of variance and Kruskal Wallis tests applied. A p-value of < 0.05 was considered as statistically significant.

## Results

The study population were 160 thalassemia patients with mean age of 21.61±7.22 ranged from 5 to 39 years and controls with mean age of 16.08 ±2.98 ranged from 11 to 24 years. Variables of AoD and AS had normal distribution (p>0.05) when all participants were considered. In the case of thalassemia participants, the variables of age, weight, AoD and AS were normal. All other variables had free distribution (table1). Amongst the variables in the study, diastolic blood pressure (test value=4478.00, p<0.001), AoD(test value=-4.036, p<0.001), AoS(test value=2413.00, p<0.001), Ferritin(test value=00.00, p<0.001), AS(test value=9.418, p<0.001), AD(test value=2890.00, p<0.001), PSEM(test value=3530.00, p<0.001) and ASβ index(test value=2256.00, p<0.001) were significantly different in groups. From these variables, AoS, AoD, ferritin, AS and AD were higher in thalassemia patients when the remained were lower (table 2). In accordance with the level of iron deposition levels grouping by MRI T2* in heart, the changes of the study parameters were compared and resulted that only ferritin were higher in those thalassemia patients who the Iron deposition was abnormal in heart (2131.89±1992.74 v.s 4887.66±3122.59) (table 3), when all the study variables were non-significant based on iron deposition level grouping by MRI T2* in liver (table 4). Thalassemia patients grouped based on the different levels of ferritin and the study variables compared in these categories and resulted that all the variables were free of significant changes except Aod(P=0.033) and AoS (P=0.011) that both were higher in the ferritin level of 3187-6051(table 5).

**Table 1:** Normality test for the study variables in all and only thalassemia participants

**All Participants**					**Thalassemia Patients**			
**Variables**	**Mean**	**SD**	**KS**	**P**	**Mean**	**SD**	**KS**	**P**
**Age**	19.76	6.67	0.12	<0.001	21.61	7.22	.06	0.200
**Wight**	44.70	11.34	0.06	0.017	43.19	10.52	.06	0.200
**Height**	151.01	13.51	0.08	0.001	150.22	13.47	.10	0.001
**SBP**	101.30	16.39	0.19	<0.001	101.66	11.69	.14	<0.001
**DBP**	67.17	9.05	0.19	<0.001	65.66	9.73	.19	<0.001
**AOD**	2.15	0.38	0.05	0.200	2.24	0.29	.07	0.032
**AOS**	2.50	0.44	0.07	0.003	2.68	0.31	.05	0.200
**Ferritin**	2142.33	2765.91	0.22	<0.001	3186.76	2864.59	.18	<0.001
**Aortic Strain**	16.68	8.43	0.04	0.200	20.00	6.75	.04	0.200
**AD**	0.01	0.01	0.07	0.007	0.01	0.01	.11	<0.001
**PSEM**	272.98	397.39	0.28	<0.001	209.85	133.28	.18	<0.001
**ASβI**	39.95	39.03	0.26	<0.001	26.88	14.16	.19	<0.001
**BMI**	19.36	3.30	0.08	0.002	18.92	3.10	.07	0.043

Systolic Blood pressure (SBP), Diastolic Blood Pressure(DBP), Aortic diameter in Diastole(AOD), Aortic diameter in Systole(AOS), Aortic distensibility (AD), Aortic stiffness β index(ASβI), Pressure strain elastic modulus(PSEM), Body Mass Index (BMI).

**Table 2:** Case-control comparison in the variables

**Variables**	**Groups**	**Mean**	**SD**	**Mean Rank Sum rank**	**Test Value**	**P- value**
**Age**	case	21.61	7.22	140.09	3265.500	<0.001
	Controls	16.08	2.98	81.32		
**Weight**	case	43.19	10.52	112.62	5138.500	.013
	Controls	47.72	12.33	136.27		
**Height**	case	150.22	13.47	117.22	5875.500	.300
	Controls	152.60	13.53	127.06		
**SBP**	case	101.66	11.69	118.12	6019.500	.442
	Controls	100.09	23.06	125.26		
**DBP**	case	65.66	9.73	108.49	4478.000	<0.001
	Controls	70.56	6.84	144.53		
**AOD**	case	2.24	0.29		-4.036	<0.001
	Controls	1.96	0.48			
**AOS**	case	2.68	0.31	145.42	2413.000	<0.001
	Controls	2.15	0.47	70.66		
**Ferritin**	case	3186.76	2864.59	160.50	0.000	<0.001
	Controls	53.45	29.94	40.50		
**TSH**	case	3.74	2.15	129.74	4922.000	.004
	Controls	2.86	1.45	102.03		
**FBS**	case	105.47	30.80	149.91	1695.000	<0.001
	Controls	82.46	10.13	61.69		
**Aortic Strain**	case	20.00	6.75		9.418	<0.001
	Controls	11.17	7.02			
**AD**	case	0.01	0.01	142.44	2890.000	<0.001
	Controls	0.01	0.01	76.63		
**PSEM**	case	209.85	133.28	102.56	3530.000	<0.001
	Controls	387.51	640.02	156.38		
**ASβI**	case	26.88	14.16	94.60	2256.000	<0.001
	Controls	65.34	55.64	172.30		
**BMI**	case	18.92	3.10		-2.957	0.003
	Controls	20.23	3.53			

Systolic Blood pressure (SBP), Diastolic Blood Pressure (DBP), Aortic diameter in Diastole (AOD), Aortic diameter in Systole (AOS), Aortic distensibility (AD), Aortic stiffness β index (ASβI), Pressure strain elastic modulus (PSEM), Thyroid stimulating hormone (TSH,) Fasting blood sugar (FBS).

**Table 3:** Comparison of the study variables in groups of thalassemia patients based on normal abnormal Iron overload in Heart

**Variables**	**MRI Heart Status**	**N**	**Mean**	**SD**	**Mean Rank**	**Sum Rank**	**Test Value**	**P- value**
**Age**	Normal	45	22.71	6.91			0.324	0.747
	Abnormal	35	22.23	6.17				
**Weight**	Normal	45	46.13	8.83			0-0.059	0.953
	Abnormal	35	46.26	9.89				
**Height**	Normal	45	153.4	10.55	40.21	1809.5	774.5	0.9
	Abnormal	35	154.23	11.15	40.87	1430.5		
**SBP**	Normal	45	101.33	9.38	40.27	1812	777	0.917
	Abnormal	35	101.29	14	40.8	1428		
**DBP**	Normal	45	66.11	8.59	42.02	1891	719	0.494
	Abnormal	35	64.29	11.19	38.54	1349		
**AOD**	Normal	45	2.22	0.27	36.59	1646.5	611.5	0.088
	Abnormal	35	2.32	0.31	45.53	1593.5		
**AOS**	Normal	45	2.62	0.28			-1.799	0.076
	Abnormal	35	2.75	0.35				
**Ferretin**	Normal	45	2131.89	1992.74	31.17	1402.5	367.5	<0.001
	Abnormal	35	4887.66	3122.59	52.5	1837.5		
**Aortic Strain**	Normal	45	18.41	7.26			-0.103	0.918
	Abnormal	35	18.57	6.88				
**AD**	Normal	45	0.01	0.01	40.64	1829	781	0.95
	Abnormal	35	0.01	0.01	40.31	1411		
**PSEM**	Normal	45	232.59	154.25	40.36	1816	781	0.95
	Abnormal	35	241.4	187.05	40.69	1424		
**ASβI**	Normal	45	30.52	17.83	41.38	1862	748	0.702
	Abnormal	35	29.77	17.75	39.37	1378		
**BMI**	Normal	45	19.54	2.96	42.02	1891	719	0.506
	Abnormal	35	19.36	3.21	38.54	1349		

Systolic Blood pressure (SBP), Diastolic Blood Pressure (DBP), Aortic diameter in Diastole (AOD), Aortic diameter in Systole (AOS), Aortic distensibility (AD), Aortic stiffness β index (ASβI), Pressure strain elastic modulus (PSEM), Body Mass Index (BMI).

**Table 4:** Comparison of the study variables in groups of thalassemia patients based on normal and abnormal iron overload in liver

**Variables**	**MRI Liver Status**	**N**	**Mean**	**SD**	**Mean Rank**	**Sum Rank**	**test Value**	**P- value**
**Age**	Normal	11	23	6.05			0.27	0.788
	Abnormal	69	22.42	6.68				
**Weight**	Normal	11	49.18	8.49			1.159	0.25
	Abnormal	69	45.71	9.33				
**Height**	Normal	11	155.91	12.47	43	473	352	0.7
	Abnormal	69	153.42	10.52	40.1	2767		
**SBP**	Normal	11	106.36	10.74	50.91	560	265	0.101
	Abnormal	69	100.51	11.54	38.84	2680		
**DBP**	Normal	11	69.09	6.25	49.86	548.5	276.5	0.138
	Abnormal	69	64.71	10.14	39.01	2691.5		
**AOD**	Normal	11	2.25	0.23	41	451	374	0.939
	Abnormal	69	2.27	0.3	40.42	2789		
**AOS**	Normal	11	2.65	0.2			-0.348	0.729
	Abnormal	69	2.68	0.33				
**Ferritin**	Normal	11	2222.27	2798.02	29.09	320	254	0.08
	Abnormal	69	3515.33	2873.82	42.32	2920		
**Aortic Strain**	Normal	11	18.34	8.84			-0.072	0.943
	Abnormal	69	18.5	6.81				
**AD**	Normal	11	0.01	0.01	42.05	462.5	362.5	0.812
	Abnormal	69	0.01	0.01	40.25	2777.5		
**PSEM**	Normal	11	347.11	379.16	38.95	428.5	362.5	0.812
	Abnormal	69	218.8	97.32	40.75	2811.5		
**ASβI**	Normal	11	40.08	38	38.73	426	360	0.785
	Abnormal	69	28.61	11.52	40.78	2814		
**BMI**	Normal	11	20.2	2.55	47.95	527.5	297.5	0.252
	Abnormal	69	19.34	3.13	39.31	2712.5		

Systolic Blood pressure (SBP), Diastolic Blood Pressure (DBP), Aortic diameter in Diastole (AOD), Aortic diameter in Systole (AOS), Aortic distensibility (AD), Aortic stiffness β index (ASβI), Pressure strain elastic modulus (PSEM), Body Mass Index (BMI).

**Table 5:** Comparison of the study variables in groups of thalassemia patients based on levels of ferritin

**Variables**	**Ferritin Groping**	**Mean**	**SD**	**Mean Rank**	**Test Value**	**P value**	**Variables**	**Mean**	**SD**	**Mean Rank**	**Test Value**	**P value**
**Age**	<3187	21.68	7.66		0.913	0.436	**AOS**	2.66	0.31		3.81	0.011
	3187-6051	22.8	6.35					2.81	0.29			
	6051-8915	20.38	6.55					2.55	0.23			
	8915	19	7.07					2.57	0.42			
**Weight**	<3187	42.01	10.44		1.572	0.198	**Aortic Strain**	20.19	6.51		0.112	0.953
	3187-6051	44.69	8.1					19.51	7.71			
	6051-8915	46.81	11.76					20.16	5.79			
	8915	41.33	15.17					19.43	8.33			
**Height**	<3187	148.16	13.75	74.39	8.269	0.041	**AD**	0.01	0.01	83.77	1.199	0.753
	3187-6051	155.66	10.73	98.84				0.01	0	75.16		
	6051-8915	152.24	11.92	84.14				0.01	0.01	77.24		
	8915	146.11	18.14	65.17				0.01	0.01	74.39		
**SBP**	<3187	101.63	11.36	79.66	1.152	0.765	**PSEM**	201.97	118.69	77.23	1.199	0.753
	3187-6051	101.71	11.75	81.67				209.12	90.28	85.84		
	6051-8915	103.57	8.96	87.4				206.96	104.77	83.76		
	8915	97.22	19.54	68.72				302.65	339.11	86.61		
**DBP**	<3187	66.05	9.53	81.68	4.454	0.216	**ASβI**	26.14	13.15	79.47	0.63	0.89
	3187-6051	65.86	9.51	81.94				27.93	12.37	85.79		
	6051-8915	66.67	10.04	85.69				25.51	9.91	78.14		
	8915	58.33	10.61	50.33				33.8	31.41	76.33		
**AOD**	<3187	2.22	0.29	78.63	8.767	0.033	**BMI**	18.88	3.05	80.98	2.348	0.503
	3187-6051	2.36	0.28	98.57				18.46	3.1	72.83		
	6051-8915	2.13	0.22	63.93				19.98	3.51	92.24		
	8915	2.16	0.36	68.61				18.66	2.5	77.83		

Systolic Blood pressure (SBP), Diastolic Blood Pressure(DBP), Aortic diameter in Diastole(AOD), Aortic diameter in Systole(AOS), Aortic distensibility (AD), Aortic stiffness β index(ASβI), Pressure strain elastic modulus(PSEM), Body Mass Index(BMI)

## Discussion

Despite the fact that the side effects of thalassemia have particularly decreased recently, the patients still experience numerous difficulties (17). At present, the most widely recognized complexity and the primary driver of death in these patients are heart failure and fatal arrhythmia (18). A change in aortic stiffness has been demonstrated in the absence of cardiac iron overload in thalassemia (19). The present study revealed that DBP, AOD, AOS, ferritin, AS, AD, PSEM and ASβ index were significantly different in thalassemia compared with the controls. From these parameters, AOD, AOS, ferritin, aortic strain and AD were higher. Several studies reported lower blood pressure, aortic strain and AD and higher ASβ index and PSEM in thalassemia compared with controls (8,9,20-22) that is in same line with our findings. It has been provided that the first side effects of thalassemia in heart are increased arterial stiffness, endothelial dysfunction, and LV hypertrophy that decrease mechanical efficiency of the heart (23). Although an association between iron loading and heart disease in transfusion-dependent thalassemia patients has been identified, a new serum ferritin level of 3000 ng / mL has been proposed to indicate an increased risk (24). Once, our groups of patients were based on the levels of ferritin and the study variables compared in these categories and resulted that all of the basic and stiffing variables had no significant changes. In this regards, Gedikliet al., (8) demonstrated that aortic elastic indices were associated with ferritin levels. Valenti et al., (25) conducted a study to evaluate the association of serum hepcidin and iron stores with PWV as a parameter of stiffness in patients with hypertension. They concluded that hyperferritinemia in thalassemia was associated with high aortic stiffness and cardiac diastolic dysfunction, and low circulating hepcidin was associated with increasing aortic stiffness. Chung et al. (14) and Ulger et al. (26) found no significant correlation between ferritin level and stiffness parameters. They also found a negative correlation of AS and AD with liver iron deposition using ferritin levels but a positive and significant correlation was observed with PSEM. Dissimilar correlation was found by Nielsen et al. (27) between aorta stiffing and ferritin. In the present study, we found that none of the stiffing parameters except AoS and AoD showed a significant correlation with ferritin levels. The difference in the results of the present and in Nielsen et al.’s (27) studies may be due the participant‘s age and the stiffness parameter. Liver iron levels are a more reliable indicator of tissue iron loading than ferritin and have been shown to have a lower correlation between hepatic iron storage and serum ferritin levels in patients receiving iron chelating agents (25-27). The aorta is not only a conduit that carries blood to tissues, but also an important regulator of the entire cardiovascular system. The association between higher levels of ferritin and increased arterial stiffness is consistent with the hypothesis that increased iron storage in the body is associated with the progression of atherosclerosis (25). Frequent blood transfusions as part of the management of thalassemia are often associated with iron overload, which can accumulate in many organs, especially the heart and liver (28). The present study found that none of stiffing parameters had significant association with iron overload in heart and liver. T2*MRI provides a fast and reproducible method to detect myocardial iron overload that occurs after a heavy transfusion load greater than 290 ml/kg red blood cell (28). Eghbali el al. (29) after investigating 60 patients with thalassemia found no significant correlation between serum ferritin level and cardiac T2*MRI but a significant correlation with liver T2*MRI. Karimi et al. (30) found that serum ferritin levels were negatively and significantly correlated with liver MRI T2 *. In a study by Wahidiyat et al., (31) the aim was to measure the iron load in the heart and liver of thalassemia using T2 * MRI. They found that the majority of subjects had normal heart iron stores. In a report by El-Shanshory et al., (32), heart MRI T2 * found 32% with iron overload in patients with thalassemia when Wahidiyatetal (31) found 70.4% had severe hepatic iron overload. From these late studies, it is inferred that. There is a significant but weak relationship between cardiac MRI T2 * and serum ferritin, and a slightly significant relationship between liver iron levels and serum ferritin. 

The reason why this occurs, is due to the fact that patients with hepatic overload generally presented earlier than cardiac overload. In addition, the pressure accumulation represented by MRI T2* appears to establish a critical relationship with serum ferritin levels.

 Cheung et al., (14) point by point extended carotid arterial stiffness, endothelial dysfunction, and left ventricular hypertrophy in thalassemia, giving the ﬁrst signal that arterial system function in thalassemia was peril by endothelial dysfunction and expanded stiffness. They proposed that the useful variety of the blood vessel tone coupled with basic modification of blood vessel divider contributed to the overall increment in systemic arterial stiffness. Kobayashi et al. (33) have regarded that iron chelating in adults with coronary artery contamination progresses endothelium-established vasodilatation. Stiffness endorses inward remodeling of small arteries, which will increase resistance, blood pressure, and in turn, important artery stiffness, hence, growing a harmful observations circle.  Chronic antihypertensive remedies can lessen stiffness past passive discounts because of reduced blood pressure. Preventive medicines such as lipid-reducing tablets and anti-diabetic medicines have extra consequences on stiffness, unbiased of pressure. Newer anti-inflammatory tablets additionally have blood pressure-unbiased consequences. Reduction of stiffness is predicted to confer advantages past the reducing of pressure, despite the fact that this assumption is not but established (30). Therefore, evaluation of aortic elastic properties as nontraditional cardiovascular risk factors may also make contributions to the identiﬁcation of future cardiovascular dangers in thalassemia. From the present study, it was discovered that ferritin became better in thalassemia with coronary heart atypical iron deposition while the complete acquire to look at parameters had been non-vast principally based totally on iron deposition in liver. From this study, it was concluded that AOS, AOD, ferritin, aortic strain and AD were higher in thalassemia when PSEM and ASβ index were lower. Ferritin was higher in those thalassemia patients whose iron deposition was abnormal in the heart. Stiffing parameters were similar in groups of thalassemia patients based on normal and abnormal iron deposition in the heart and the liver. We can recommend hepatic and cardiac MRI T2* in addition to measurement of serum ferritin for better evaluation of patients with major thalassemia.
